# Pre-Analytical Variables in Digestive Cancer Pathology: A Systematic Assessment of Morphological Preservation in Tumoral and Normal Tissues

**DOI:** 10.3390/diagnostics16030445

**Published:** 2026-02-01

**Authors:** Lydia el Moutaoukkil, Laila Chbani, Imane Toughrai, Bachir Benjelloun

**Affiliations:** 1 Pathology Department, University Hospital Hassan II, Fes 30050, Morocco; lydiaem26@gmail.com; 2 Visceral Surgery Department, University Hospital Hassan II, Fes 30050, Morocco; imane.toughrai@usmba.ac.ma (I.T.);

**Keywords:** fixation, cold ischemia, morphology

## Abstract

**Background/Objectives**: This research covers both tumoral and non-tumoral (adjacent normal) tissues. Non-tumoral tissue samples were obtained from surgical resection margins located at least 5 cm from the tumor edge, with histological confirmation of the absence of tumor involvement. **Methods**: These samples, varying from 0 weeks to 1 week, were systematically evaluated. The assessment encompassed critical histological aspects such as tissue architecture, nuclear morphology, cytoplasmic features, and membrane characteristics. A scoring system comprising three categories (good, fair, and bad) was employed to gauge the extent of morphological alterations observed in tissue specimens. Statistical analyses were conducted using the “IBM SPSS Statistics 26.0” software. **Results**: Our findings unveiled a statistically significant association between tissue type and morphological degradations, highlighting the impact of prolonged cold ischemia time and fixation time on cellular swelling, cellular integrity loss, and tissue architecture disruption. The correlation between normal and tumor tissue was statistically significant for pre-analytical parameters evaluated with a strong influence on tumor tissue in cold ischemia time with a *p* = 0.046, *p* = 0.020, *p* = 0.029. For fixation times, the impact was significant for most of the morphological parameters, *p* = 0.021, *p* = 0.005, *p* = 0.023. **Conclusions**: These observations underscore the critical importance of minimizing cold ischemia time and refining fixation protocols to uphold tissue morphology, protein and molecular integrity. Such endeavors are pivotal in ensuring accurate histopathological evaluation and facilitating precise molecular analyses in the context of digestive cancer research.

## 1. Introduction

Gastrointestinal cancers are a group of cancers that affect various organs of the digestive system, including the esophagus, stomach, liver, pancreas, colon, and rectum. These cancers are among the most common in the world [1,2]. Morphology analysis is crucial for determining the tissue prognostic factors in digestive cancer, so using a partial qualitative approach will help us to classify the cancer’s tissue-based intensity and to understand the tumor’s potential severity, estimating the patient’s future health trajectory [3]. However, beyond the realm of microscopic examination lies a critical phase that holds the potential to significantly influence the accuracy and reliability of histopathological assessments–the pre-analytical phase [4]. One of the pivotal pre-analytical factors influencing morphological analysis is the effect of cold ischemia and fixation time [5,6]. The duration between organ excision and its preservation can exert substantial changes on cellular structures [7].

In gastrointestinal tumors specifically, previous studies have demonstrated organ-specific effects of pre-analytical variables. Portier et al. showed that delayed fixation in breast cancer can alter HER2 detection, with similar observations reported for colorectal cancer markers by Fan et al. [8]. In pancreatic tissue, the high enzymatic content makes it particularly susceptible to autolysis during cold ischemia, as demonstrated by Koonmee et al. in cholangiocarcinoma specimens [9]. Liu et al. reported that renal cell carcinoma showed significant gene expression changes after 1 h of cold ischemia [10]. Despite these organ-specific studies, a comprehensive comparative assessment across multiple digestive organs remains limited

This study addresses the pivotal question of how cold ischemia and fixation time impact the morphological and histological parameters of digestive cancer tissues [9,10]. By systematically exploring these variables across diverse organs, from the liver to the rectum, this research elucidates the specific effects on both tumoral and normal tissues [8]. The findings not only underscore the significance of optimized pre-analytical practices [11] but also contribute to the broader discourse on enhancing the accuracy of histopathological diagnoses in cancer [12].

The aim of our study is to elucidate the impact of cold ischemia and fixation time on the morphological integrity of tumoral and normal tissues in digestive cancer [13]. Specifically, we aim to assess how these two key pre-analytical variables influence the preservation quality of various morphological parameters, including morphology, cytoplasmic details, nucleic details, and membrane. By comparing the effects on tumoral versus normal tissues, our study seeks to provide critical insights into the optimal time handling and processing protocols for these tissue types.

## 2. Materials and Methods

### 2.1. Ethical Aspects

This study was approved by the research ethics committee at the University Hospital Center Hassan II, Morocco, n°21̸22, date 8 December 2022. Informed consent was obtained from all subjects involved in the study.

#### 2.1.1. *Study Design and Population*

**Figure 1:**
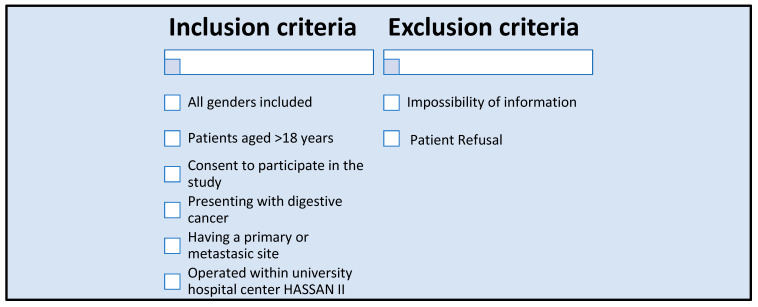
Patient criteria inclusion.

Histopathological subtypes were classified according to WHO criteria, with adenocarcinomas representing 85% of cases (Table 1). All specimens were obtained from primary tumor resections; metastatic specimens were excluded to ensure biological homogeneity (Figure 1).

#### 2.1.2. Specimen Collection and Processing

The mastery of the pre-analytical phase is initiated by monitoring the operative specimens taken from patients hospitalized in the visceral surgery departments A and B with digestive cancer.

**Figure 2:**
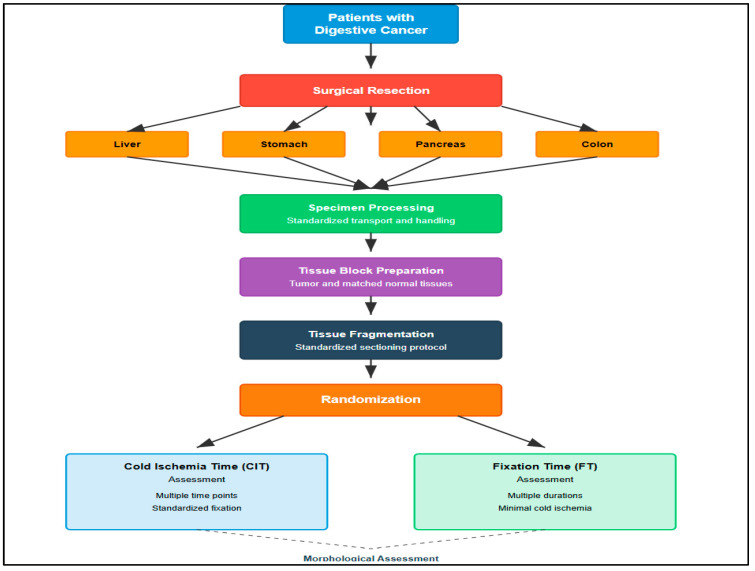
Experimental Design and Sample Processing Workflow.

A total of 72 specimens were conveyed post-resection. The experimental design and sample processing workflow are illustrated in Figure 2. 

Specimens were maintained at an ambient temperature (20–25 °C) in sealed containers lined with saline-moistened gauze to prevent desiccation, with transport time from surgery room to pathology department standardized at <10 min. Cold ischemia timing began at the point of vascular clamping. For extended CIT intervals (24–96 h), tissue cubes were stored in sealed specimen containers at ambient temperature under controlled humidity conditions to model delayed fixation scenarios that may occur in resource-limited settings or during specimen transport. Upon receipt, specimens were immediately bread-loafed at 5 mm intervals to ensure adequate fixative penetration.

Following grossing, both tumor and normal specimens were sectioned into standardized tissue cubes (0.5 × 0.5 × 0.5 cm) following sterile procedures. Importantly, CIT intervals were applied to these standardized tissue cubes post-grossing (not to intact specimens), thereby ensuring uniform tissue dimensions and consistent diffusion dynamics across all experimental conditions [14,15].

Randomization and blinding procedures: Tissue fragments were randomly assigned to different CIT and FT conditions using a computer-generated randomization sequence. The primary pathologist (L.C.) performing morphological assessment was blinded to the experimental conditions (CIT and FT duration) through coded slide labeling; however, true blinding to tissue type (tumor versus normal) was not feasible given the readily apparent histological differences on H&E examination. To ensure reliability, a second independent pathologist performed scoring on 30% of randomly selected samples. Inter-rater reliability was calculated using Cohen’s kappa coefficient.

The duration of fixation and cold ischemia time has been routinely recorded for all specimens following the pre-analytical guidelines established by the College of American Pathologists (CAP) and the International Society for Biological and Environmental Repositories (ISBER) https://www.cancerresearch.org/.

We chose the following protocol as detailed in [16]: 72 tumoral and healthy tissues were selected according to five cold ischemia times (<1 h (CIT-1), 6 h (CIT-2), 24 h (CIT-3), 48 h (CIT-4), and 96 h (CIT-5)) and four fixation duration times (24 h (FT-1), 48 h (FT-2), 96 h (FT-3), and 7 days (FT-4)).

We selected nine fragments of both tumoral and normal tissues from each organ to ensure a robust comparison. Of these nine fragments, five were allocated to the cold ischemia experiment and four to the fixation time experiment, yielding a total of 20 fragments per tissue type for CIT assessment (5 timepoints × 4 organs) and 16 fragments per tissue type for FT assessment (4 timepoints × 4 organs). The fragments were divided into two distinct categories based on the processing times post-resection: cold ischemia time and fixation time.

For the cold ischemia time assessment, we used a total of five fragments per tissue type, each subjected to a different duration of ischemia to determine the impact of variable ischemic intervals on tissue integrity. These intervals were as follows: <1 h, 6 h, 24 h, 48 h, and 96 h. Notably, following the ischemic period, these fragments were then uniformly fixed for a duration of 24 h to standardize the subsequent histological analysis.

In contrast, the fixation time category involved four fragments per tissue type. These fragments were fixed for varying lengths of time to evaluate the effect of fixation duration on tissue morphology and molecular composition. The fixation times were 24 h, 48 h, 96 h, and 7 days. It is crucial to mention that these particular fragments were exposed to cold ischemia for less than one hour before fixation to minimize the ischemic interval’s confounding effects.

### 2.2. Slides Preparation

Following this, the samples were embedded in paraffin and sliced. Every slide was then examined using HES (Hematoxyline Eosine Safran) staining and sent to the pathologist of the platform to identify an area containing more than 50% tumor cells, on which a histological study will be carried out. Morphological changes in H&E-stained tissue for each fixation and duration of cold ischemia time were analyzed at ×200 magnification.

The specimens used were collected after obtaining informed consent from the patients and immediately anonymized. The study protocol was approved by the Scientific Committee of Research at CHU HASSAN II, Fes.

#### Impact Scoring System

We propose a scoring system based on the degree of alteration observed in tissue morphology to evaluate the impact of cold ischemia time and delayed fixation on morphological parameters (Table 2). The scoring system will consist of three categories: good (2), fair (1), and bad (0), based on the extent of morphological alteration observed in tissue specimens [17].

### 2.3. Statistical Analysis

Statistical analyses were performed using SPSS 26.0.0 (SPSS Inc., Chicago, IL, USA). The primary endpoint was the global morphology score, calculated as the arithmetic mean of the four component scores (morphology, cytoplasmic details, nuclear details, and membrane integrity), with possible values ranging from 0 to 2. To account for the non-independence of multiple fragments derived from the same patient, ordinal logistic regression with mixed-effects modeling (patient as random effect) adjusted for organ type was used to assess associations between tissue type (tumor vs. normal) and morphological degradation. Cluster-robust standard errors were employed to ensure valid inference.

Descriptive data were presented as means with standard deviations. Associations between tumor (assessing as a categorical variable) and pre analytical factors of tumor were assessed using the χ^2^-test and Pearson’s correlation. Comparisons among morphological types and rates of tumoral and normal tissues expression were performed with Pearson’s chi-square. Results with a *p* value less than 0.05 (*p* ≤ 0.05) were accepted as significant.

## 3. Results

Inter-rater reliability assessment showed substantial agreement between pathologists (κ = 0.78, 95% CI: 0.71–0.85).

Our study carefully analyzed tissue samples from a diverse group of patients who underwent surgical resection for gastrointestinal cancers, including liver, stomach, pancreatic, and colon cancers within our university Hospital Hassan II. We evaluated a total of 72 samples, which were aliquoted to examine tumor tissue and normal tissue. The purpose of this comprehensive collection is to provide a robust framework to evaluate the morphological impact of preanalytical variables, namely cold ischemia time (CIT) and fixation time (FT).

**Organ-specific susceptibility analysis:** When stratified by organ type, differential susceptibility to cold ischemia was observed. Pancreatic tissue showed the greatest sensitivity (50% degradation at 24 h), followed by liver (40% at 24 h), stomach (35% at 24 h), and colon (30% at 24 h). These differences were statistically significant (*p* = 0.012 for organ-type interaction).

**Figure 3:**
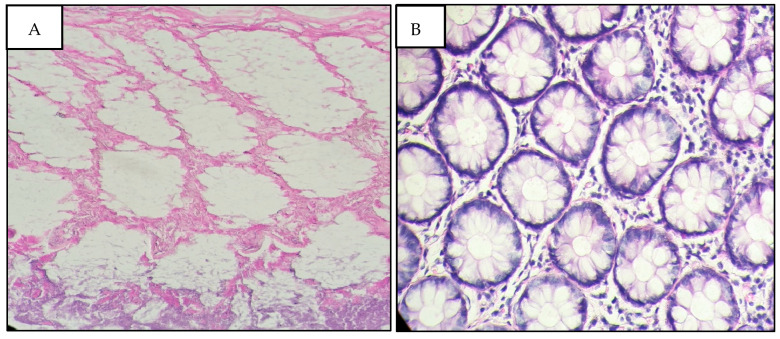
Colonic mucosa showing architectural preservation with clearly visible cytonuclear details for cold ischemia of less than 1 h (**A**). Ghostly appearance of the epithelium with disappearance of cytonuclear details of the overall morphology for 96 h of cold ischemia (**B**). H&E staining, ×200 magnification, scale bar = 50 μm.

**Figure 4:**
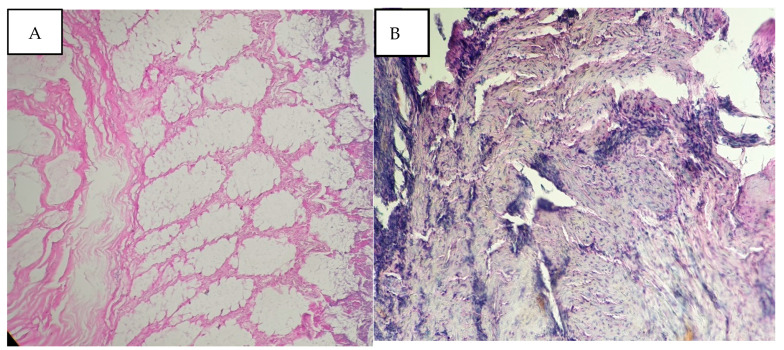
Preservation of the general architecture with a recognizable appearance of the healthy mucosa for 96 h of cold ischemia (**A**); the architectural alteration is more marked making morphological analysis impossible for the tumor tissue after 96 h of cold ischemia (**B**). H&E staining, ×200 magnification, scale bar = 50 μm.

Utilizing Pearson’s chi-square test and the computation of *p*-values, our results highlighted a significant correlation between extended CIT and notable morphological degradation in both tumoral and normal tissues. Specifically, tissues subjected to CIT beyond 24 h exhibited pronounced cellular swelling and architectural disarray, underscoring the urgency of minimizing ischemia duration post-resection (Figure 3). At extended cold ischemia times (96 h), normal tissues showed relative preservation of general architecture, while tumoral tissues exhibited marked alterations rendering morphological analysis impossible (Figure 4). Similarly, variations in FT revealed that overly prolonged fixation could induce artifacts, such as nuclear smudging and cytoplasmic vacuolization, potentially obscuring critical diagnostic details.

**Figure 5:**
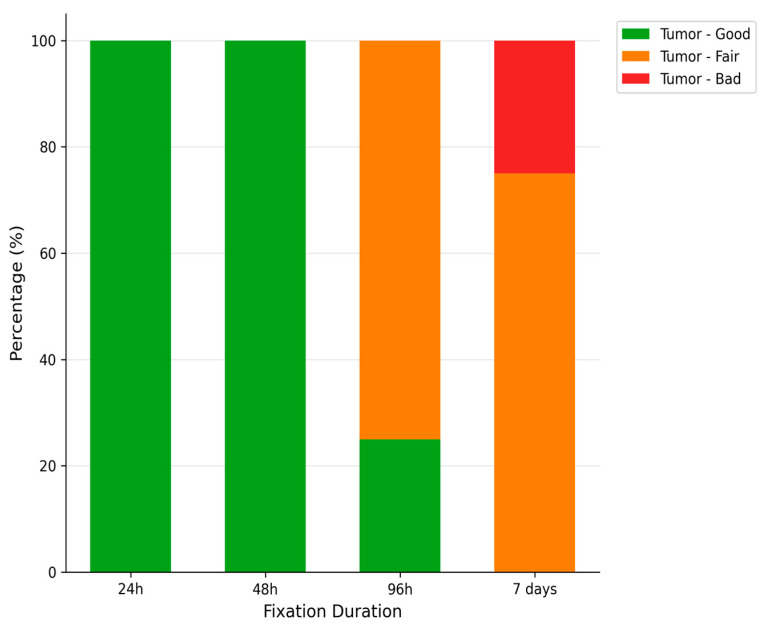
Impact of Fixation Time on Tissue Morphology.

Impact of fixation time and cold ischemia on morphological parameters:

Fixation Time Score:

The impact of fixation duration on tumoral tissue morphology is illustrated in Figure 5.

**Description:** Effect of fixation duration on morphological quality. Extended fixation beyond 96 h significantly compromises tissue quality.

A total of 16 fragments of each type of tissue were analyzed under different fixation durations, revealing significant alterations in tissue preservation quality attributable to the length of the fixation period.

For tumoral tissues, the analysis demonstrated a clear trend of diminishing morphological integrity with prolonged fixation times. Initially, all samples maintained their morphology at the shortest fixation time (24 h). However, as fixation extended, a decline in morphological quality was observed, with the longest fixation time showing a notable decrease in the preservation of morphology, cytoplasmic details, and nucleic integrity. Specifically, at the maximum fixation duration, 25% of tumoral tissue samples retained good morphology, while 75% exhibited only fair preservation. The statistical analysis confirmed these observations as significant, with a *p*-value of 0.023, underscoring the detrimental effects of extended fixation on tissue morphology.

Similarly, normal tissues displayed a parallel decline in preservation quality with increased fixation times. The degradation of morphological and cytoplasmic details became more pronounced at extended fixation durations, highlighting a heightened sensitivity of normal tissues to the fixation process. The most extended fixation period resulted in a significant reduction in the quality of membrane integrity and nucleic details, particularly impacting normal tissues more severely than tumoral tissues. This was statistically significant, with *p*-values of 0.005 for morphology and 0.004 for cytoplasmic details, indicating a profound impact of fixation time on the preservation of normal tissue integrity.

Tumoral tissue:

Morphology *p* = 0.023:

MORPHOLOGYTotal012fixation time1Effectif0044% fixation T0.0%0.0%100.0%100.0%2Effectif0044% fixation T0.0%0.0%100.0%100.0%3Effectif0314% fixation T0.0%75.0%25.0%100.0%4Effectif1304% fixation T25.0%75.0%0.0%100.0%TotalEffectif16916% fixation T6.3%37.5%56.3%100.0%

Cytoplasmic details *p* = 0.023:

Cytoplasmic detailsTotal012Fixation time1Effectif0134% fixation T0.0%25.0%75.0%100.0%2Effectif0404% fixation T0.0%100.0%0.0%100.0%3Effectif1304% fixation T25.0%75.0%0.0%100.0%4Effectif2204% fixation T50.0%50.0%0.0%100.0%TotalEffectif310316% fixation T18.8%62.5%18.8%100.0%

Membrane *p* = 0.175:

MEMBRANETotal012Fixation time1Effectif0134% fixation T0.0%25.0%75.0%100.0%2Effectif1124% fixation T25.0%25.0%50.0%100.0%3Effectif1304% fixation T25.0%75.0%0.0%100.0%4Effectif2204% fixation T50.0%50.0%0.0%100.0%TotalEffectif47516% fixation T25.0%43.8%31.3%100.0%

Nucleic Details *p* = 0.021:

NUCLEAR DETAILSTotal012 Fixation time1Effectif0134% fixation T0.0%25.0%75.0%100.0%2Effectif0224% fixation T0.0%50.0%50.0%100.0%3Effectif2204%fixation T50.0%50.0%0.0%100.0%4Effectif4004% fixation T100.0%0.0%0.0%100.0%TotalEffectif65516% fixation T37.5%31.3%31.3%100.0%

Normal tissue:

Morphology *p* = 0.005:

MORPHOLOGYTotal012Fixation S1Effectif0044% dans fixation S0.0%0.0%100.0%100.0%2Effectif0314% dans fixation S0.0%75.0%25.0%100.0%3Effectif0404% dans fixation S0.0%100.0%0.0%100.0%4Effectif2204% dans fixation S50.0%50.0%0.0%100.0%TotalEffectif29516% dans fixation S12.5%56.3%31.3%100.0%

Cytoplasmic details *p* = 0.004:

CYTOPLASMIC DETAILSTotal012Fixation S1Effectif0044% dans fixation S0.0%0.0%100.0%100.0%2Effectif1124% dans fixation S25.0%25.0%50.0%100.0%3Effectif0404% dans fixation S0.0%100.0%0.0%100.0%4Effectif3104% dans fixation S75.0%25.0%0.0%100.0%TotalEffectif46616% dans fixation S25.0%37.5%37.5%100.0%

Membrane *p* = 0.001:

MEMBRANETotal012Fixation S1Effectif0134% dans fixation S0.0%25.0%75.0%100.0%2Effectif0404% dans fixation S0.0%100.0%0.0%100.0%3Effectif0404% dans fixation S0.0%100.0%0.0%100.0%4Effectif3104% dans fixation S75.0%25.0%0.0%100.0%TotalEffectif310316% dans fixation S18.8%62.5%18.8%100.0%

Nucleic Details *p* = 0.001:

NUCLEIC DETAILSTotal012Fixation S1Effectif0044% dans fixation S0.0%0.0%100.0%100.0%2Effectif0314% dans fixation S0.0%75.0%25.0%100.0%3Effectif0404% dans fixation S0.0%100.0%0.0%100.0%4Effectif3104% dans fixation S75.0%25.0%0.0%100.0%TotalEffectif38516% dans fixation S18.8%50.0%31.3%100.0%

Comparative Analysis between Tumoral and normal Tissue.

The comparative analysis underscores the significant impact of fixation time on both tumoral and normal tissues, with each histological parameter revealing different sensitivities across tissue types. While some parameters showed balanced effects, others indicated a slight predisposition for tumoral or tissues to exhibit more pronounced alterations.


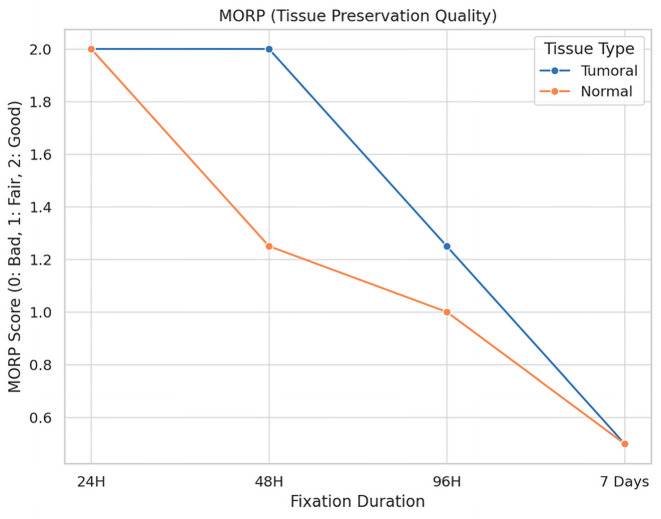



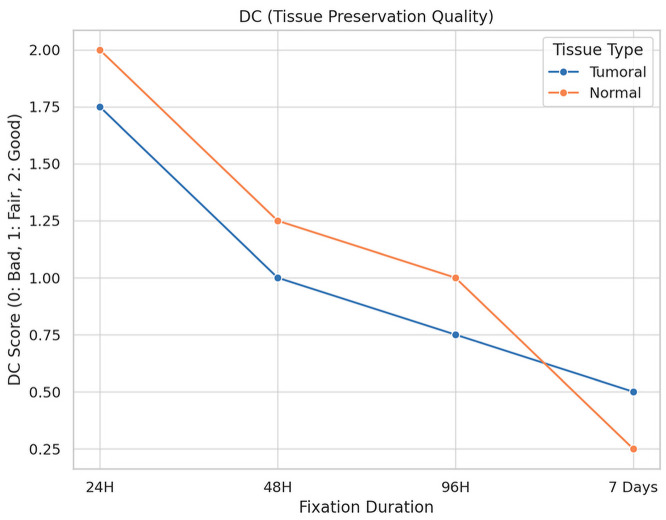



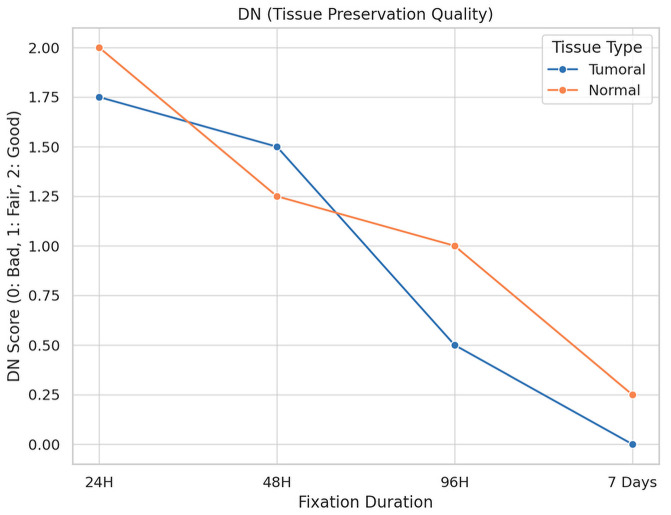


*p* = 0.319

NatureTotal01MORP0Effectif224% dans MORP50.0%50.0%100.0%1Effectif5914% dans MORP35.7%64.3%100.0%2Effectif9514% dans MORP64.3%35.7%100.0%TotalEffectif161632% dans MORP50.0%50.0%100.0%


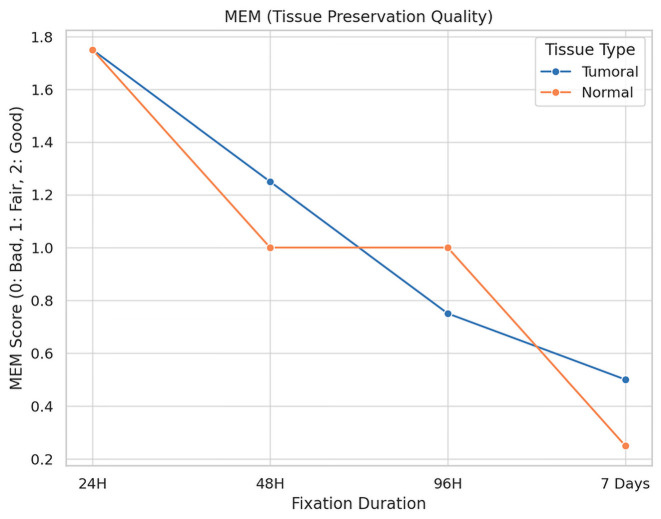


*p* = 0.343
DC0Effectif347% dans DC42.9%57.1%100.0%1Effectif10616% dans DC62.5%37.5%100.0%2Effectif369% dans DC33.3%66.7%100.0%TotalEffectif161632% dans DC50.0%50.0%100.0%DN0Effectif639% dans DN66.7%33.3%100.0%1Effectif5813% dans DN38.5%61.5%100.0%2Effectif5510% dans DN50.0%50.0%100.0%TotalEffectif161632% dans DN50.0%50.0%100.0%MEM0Effectif437% dans MEM57.1%42.9%100.0%1Effectif71017% dans MEM41.2%58.8%100.0%2Effectif538% dans MEM62.5%37.5%100.0%TotalEffectif161632% dans MEM50.0%50.0%100.0%

**Figure 6:**
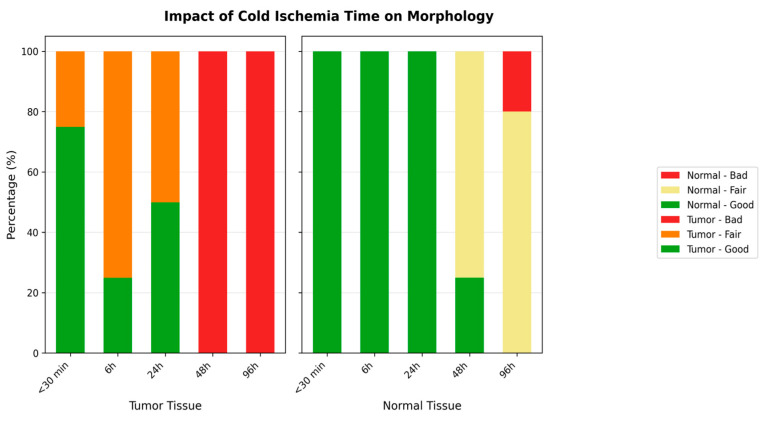
Impact of Cold Ischemia Time on Tissue Morphology. Description: Comparison of morphological degradation between tumor and normal tissues according to cold ischemia time. Tumor tissues show faster and more severe degradation. Score: 2 = Good, 1 = Fair, 0 = Bad.

Cold ischemia time score:

The impact of cold ischemia time on tissue morphology is illustrated in Figure 6.

Normal tissue:

Morphology results demonstrated 100% preservation at the shortest cold ischemia times, with a notable decline as ischemia time increased (Figure 6). At the longest ischemia times, all cases showed significant morphological degradation, for cytoplasmic details: Initially maintained well, but showed a decline with longer ischemia times, indicating a decrease in preservation quality.

Both Nucleic Details and Membrane Integrity experienced a reduction in quality with extended ischemia times.

Morphology *p* = 0.0001:

MORPTotal012Ischemie F1Effectif0044% cold Ischemia0.0%0.0%100.0%100.0%2Effectif0404% cold Ischemia0.0%100.0%0.0%100.0%3Effectif0404% cold Ischemie 0.0%100.0%0.0%100.0%4Effectif3104% cold Ischemia75.0%25.0%0.0%100.0%5Effectif4004% cold Ischemia100.0%0.0%0.0%100.0%TotalEffectif79420% cold Ischemia35.0%45.0%20.0%100.0%

Cytoplasmic details *p* = 0.176:

DCTotal012Ischemie F1Effectif0224% cold Ischemie 0.0%50.0%50.0%100.0%2Effectif0404% cold Ischemie 0.0%100.0%0.0%100.0%3Effectif1124% cold Ischemie 25.0%25.0%50.0%100.0%4Effectif1304% cold Ischemie 25.0%75.0%0.0%100.0%5Effectif2204% cold Ischemie 50.0%50.0%0.0%100.0%TotalEffectif412420% cold Ischemie 20.0%60.0%20.0%100.0%

Nucleic details *p* = 0.040:

DNTotal012Ischemie F1Effectif0134% dans Ischemie F0.0%25.0%75.0%100.0%2Effectif0314% dans Ischemie F0.0%75.0%25.0%100.0%3Effectif0224% dans Ischemie F0.0%50.0%50.0%100.0%4Effectif1304% dans Ischemie F25.0%75.0%0.0%100.0%5Effectif3104% dans Ischemie F75.0%25.0%0.0%100.0%TotalEffectif410620% dans Ischemie F20.0%50.0%30.0%100.0%

Membrane *p* = 0.038:

MEMTotal012Ischemie F1Effectif0044% dans Ischemie F0.0%0.0%100.0%100.0%2Effectif0314% dans Ischemie F0.0%75.0%25.0%100.0%3Effectif0224% dans Ischemie F0.0%50.0%50.0%100.0%4Effectif1304% dans Ischemie F25.0%75.0%0.0%100.0%5Effectif2204% dans Ischemie F50.0%50.0%0.0%100.0%TotalEffectif310720% dans Ischemie F15.0%50.0%35.0%100.0%

Tumoral tissue:

Morphology showed a substantial decline in preservation as ischemia time increased, with the longest times showing the most pronounced effects. Cytoplasmic and nucleic details echoed the trends seen in morphology, with a significant decline observed in the quality of preservation, Membrane integrity is also affected by prolonged ischemia, indicating a clear correlation between increased cold ischemia time and decreased tissue integrity.

Morphology *p* = 0.003:

MORPTotal012Ischemie F1Effectif0134% dans Ischemie F0.0%25.0%75.0%100.0%2Effectif1304% dans Ischemie F25.0%75.0%0.0%100.0%3Effectif2204% dans Ischemie F50.0%50.0%0.0%100.0%4Effectif4004% dans Ischemie F100.0%0.0%0.0%100.0%5Effectif4004% dans Ischemie F100.0%0.0%0.0%100.0%72 TotalEffectif116320% dans Ischemie F55.0%30.0%15.0%100.0%

Nucleic details *p* = 0.005:

DCTotal012Ischemie F1Effectif0134% dans Ischemie F0.0%25.0%75.0%100.0%2Effectif0314% dans Ischemie F0.0%75.0%25.0%100.0%3Effectif3104% dans Ischemie F75.0%25.0%0.0%100.0%4Effectif4004% dans Ischemie F100.0%0.0%0.0%100.0%5Effectif4004% dans Ischemie F100.0%0.0%0.0%100.0%TotalEffectif115420% dans Ischemie F55.0%25.0%20.0%100.0%

Cytoplasmic details *p* = 0.0001:

DNTotal012Ischemie F1Effectif0044% dans Ischemie F0.0%0.0%100.0%100.0%2Effectif0314% dans Ischemie F0.0%75.0%25.0%100.0%3Effectif4004% dans Ischemie F100.0%0.0%0.0%100.0%4Effectif4004% dans Ischemie F100.0%0.0%0.0%100.0%5Effectif4004% dans Ischemie F100.0%0.0%0.0%100.0%TotalEffectif123520% dans Ischemie F60.0%15.0%25.0%100.0%

Membrane *p* = 0.005:

MEMTotal012Ischemie F1Effectif0134% dans Ischemie F0.0%25.0%75.0%100.0%2Effectif0314% dans Ischemie F0.0%75.0%25.0%100.0%3Effectif3104% dans Ischemie F75.0%25.0%0.0%100.0%4Effectif4004% dans Ischemie F100.0%0.0%0.0%100.0%5Effectif4004% dans Ischemie F100.0%0.0%0.0%100.0%TotalEffectif115420% dans Ischemie F55.0%25.0%20.0%100.0%

Comparative Analysis Between Tumoral and Normal Tissue

The comparative analysis between tumoral and normal tissues underlines a negative impact of extended cold ischemia times across all examined parameters. The analysis suggests a nuanced difference in the degree of susceptibility, with a more pronounced vulnerability in tumoral tissues to the deleterious effects of prolonged cold ischemia.


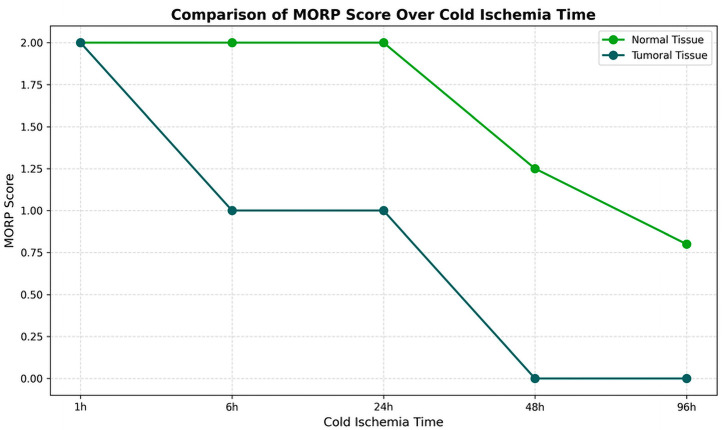



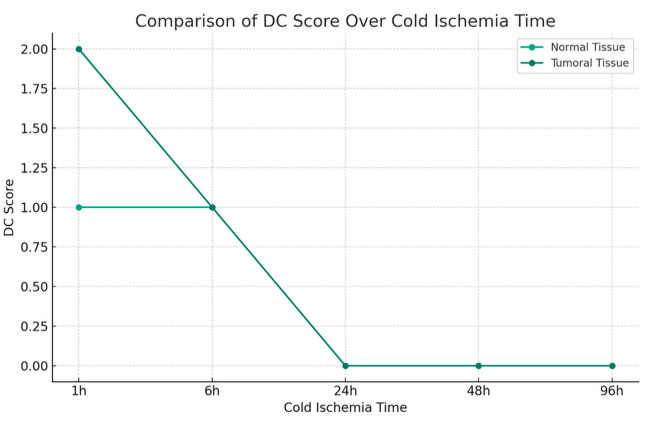



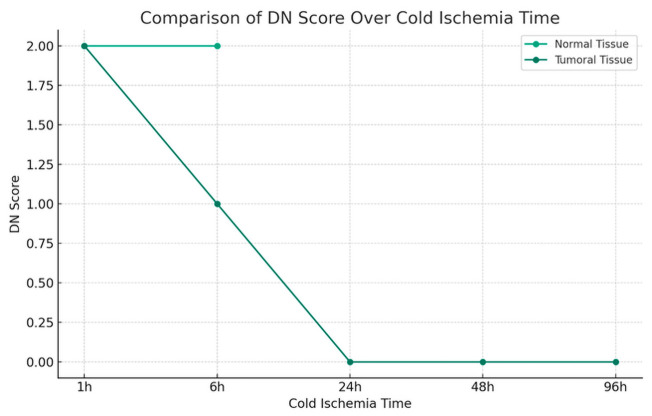



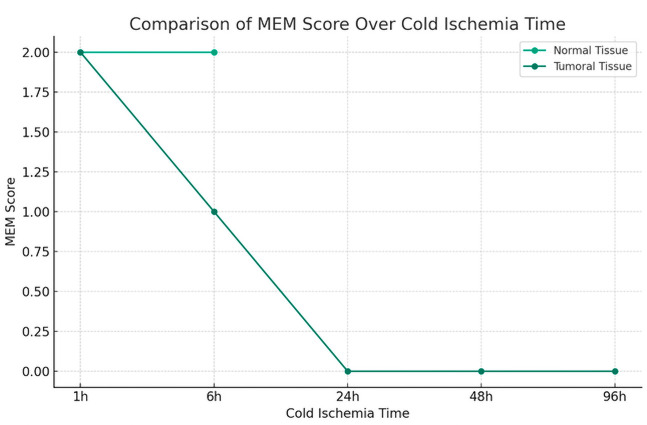


*p* = 0.442

NatureTotal01MORP0Effectif11718% dans MORP61.1%38.9%100.0%1Effectif6915% dans MORP40.0%60.0%100.0%2Effectif347% dans MORP42.9%57.1%100.0%TotalEffectif202040% dans MORP50.0%50.0%100.0%

*p* = 0.046
DC0Effectif11415% dans DC73.3%26.7%100.0%1Effectif51217% dans DC29.4%70.6%100.0%2Effectif448% dans DC50.0%50.0%100.0%TotalEffectif202040% dans DC50.0%50.0%100.0%

*p* = 0.020
DN0Effectif12416% dans DN75.0%25.0%100.0%1Effectif31013% dans DN23.1%76.9%100.0%2Effectif5611% dans DN45.5%54.5%100.0%TotalEffectif202040% dans DN50.0%50.0%100.0%

*p* = 0.029
MEM0Effectif11314% dans MEM78.6%21.4%100.0%1Effectif51015% dans MEM33.3%66.7%100.0%2Effectif4711% dans MEM36.4%63.6%100.0%TotalEffectif202040% dans MEM50.0%50.0%100.0%

**Figure 7:**
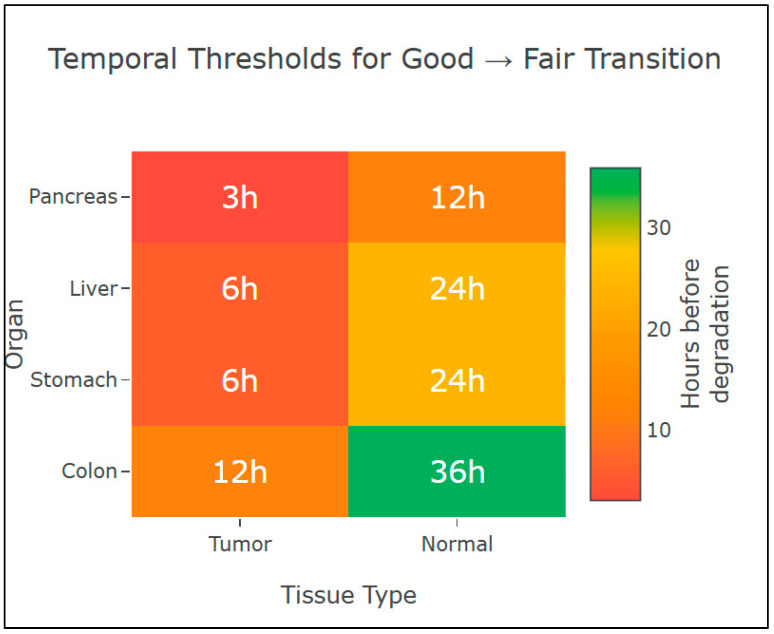
Heat map of temporal thresholds for morphological transition from good to fair quality. Values indicate hours before significant degradation occurs. Darker colors indicate more rapid degradation.

The temporal thresholds for morphological transition from good to fair quality across organs and tissue types are summarized in Figure 7, highlighting earlier degradation in tumoral tissues compared with normal tissues.

## 4. Discussion

The histological comparison in Figure 1 of colonic tissue is a striking example of the impact of ischemia on tissue composition. In the first illustration, the colonic tissue (A) exposed to less than one hour of cold ischemia shows remarkable architectural conservation. The cellular and nuclear components are resolved and distinguishable, which suggests that the limited duration of ischemia maintains the histological quality of the epithelium. This preservation is important for accurate pathological analysis, as it ensures the cytonuclear properties crucial to diagnosing and grading colonic diseases. Illustration (B) demonstrates the colonic mucosa subjected to 96 h of cold ischemia, which reveals an important degradation of tissue quality. The epithelium shows a loss of nuclear details, but the overall architecture is still recognizable. This morphological alteration that occurs following a long period of cold ischemia could have a significant impact on the accuracy of the diagnosis, the loss of cellular detail would significantly alter the cytopathological features.

In our second figure, this pair of histological images demonstrates the divergent effects of prolonged cold ischemia on normal and tumoral tissues. In (A), we observe normal tissue that has been subjected to 96 h of cold ischemia. Remarkably, despite the extended ischemic interval, the general architecture is slightly preserved, with the normal mucosa maintaining a recognizable appearance.

In stark contrast, (B) reveals tumoral tissue that has undergone the same duration of cold ischemia. Here, the impact is dramatically different, the architectural alteration is markedly pronounced, disrupting the morphological landscape to the extent that a proper analysis becomes infeasible. The tissue exhibits significant degradation, with the tumor architecture losing its definition and clarity. This pronounced distortion in tumoral tissue morphology underlines the heightened vulnerability of cancerous tissues to cold ischemia.

**Differential vulnerability of tumor versus normal tissue:** Our findings reveal that tumor tissues exhibit a 2–3-fold-higher susceptibility to pre-analytical degradation compared to normal tissues. This differential can be attributed to several factors. Tumor tissues typically display altered metabolic states with increased glycolytic activity, higher cellular density, and disrupted vascular architecture, rendering them more prone to ischemic damage. The accelerated proliferative rate and aberrant cell cycle regulation in tumor cells likely contribute to their heightened sensitivity. In contrast, normal tissues maintained better structural integrity at intermediate time points (24–48 h), suggesting inherent protective mechanisms including more efficient autophagy pathways and better preservation of cellular homeostasis under stress conditions.

**Organ-specific considerations in digestive cancers:** The observed organ-specific variations in cold ischemia susceptibility have important clinical implications. Pancreatic tissue demonstrated the most rapid degradation, with significant morphological changes occurring within 3 h for tumor tissue. This heightened sensitivity likely results from the high content of digestive enzymes (trypsin, lipase, amylase) that accelerate autolysis. Liver tissue showed intermediate resistance despite its metabolically active nature, possibly due to its regenerative capacity and robust hepatocyte architecture. Colonic tissue exhibited the greatest resistance, which may reflect evolutionary adaptation to harsh luminal conditions and the protective mucin layer. These findings support the implementation of organ-specific processing protocols rather than universal guidelines.

While normal tissues may exhibit a degree of tolerance to extended ischemic conditions, tumoral tissues demand prompt and precise handling to ensure accurate histopathological evaluation. These findings advocate for the importance of optimizing tissue handling protocols and emphasize the value of rapid tissue processing as a determinant of diagnostic accuracy in oncopathology.

Morphological parameters were greatly influenced by both pre-analytical variables, cold ischemia, and fixation time of tumoral and normal tissues. In this case, the morphological parameter in tumoral tissues significantly changed with a *p*-value of 0.023 for increased fixation time and a *p*-value of 0.0001 for cold ischemia time as markers demonstrating the statistical significance of these particular parameters on this pre-analytical variable. Similarly, the cytoplasmic details parameter in normal tissues also exhibited significant variations with a *p*-value of 0.004, supporting that these conditions had high sensitivity. The data for the nucleic acid parameter showed a significant cold ischemia response for normal tissues with a *p*-value of 0.040. This may indicate an ischemia-related cutoff level in terms of modification. Moreover, the membrane staining parameter displayed a high degree of sensitivity for tumoral tissues with a *p*-value of 0.005, suggesting that the fixative was one of the major influencing factors. Both cold ischemia and fixation time cause these marked morphological differences in tumoral as well as in normal digestive cancer tissues, as corroborated by low *p*-values. As the *p*-values are less than 0.05, it means that the difference observed is not likely due to random chance, but could be real and quantifiable data for cold ischemia and fixation time influencing both tumoral and normal digestive cancer tissue’s histopathologic attributes. This statistical validation of our findings adds weight to the undeniable significance of pre-analytical procedure optimization in diagnosing cancers through histopathological examination.

In tumoral tissues, a typical pattern of response was established to cold ischemia. In comparison with normal tissues, these tissues often showed lower scores in important morphological criteria, implying that their tolerance level towards detrimental impacts related to prolonged cold ischemia is likely to be elevated. Specifically, low global morphology scores were observed for tumoral tissues indicating an impaired overall tissue morphology state.

The divergence in tissue response was further underscored by the analysis of DC (Cytoplasmic Details) and DN (Nuclear Details) scores. Tumoral tissues were characterized by a higher occurrence of lower scores with a *p*-value of 0.046 and 0.020, reflecting the adverse impact of cold ischemia on cytoplasmic and nuclear details, critical factors for accurate tissue characterization, and cancer grading. Conversely, normal tissues demonstrated a propensity for higher DC and DN scores, indicating a relative resilience to cold ischemia.

The impact of cold ischemia was also evident in the Membrane Integrity scores. Tumoral tissues exhibited more frequent lower scores with a *p*-value of 0.029. This finding is particularly crucial considering the role of membrane integrity in various immunohistochemical analyses, where membrane proteins serve as key diagnostic and prognostic markers.

For fixation times, the morphological parameters in tumoral tissues frequently manifested lower scores, which may suggest a heightened sensitivity of tumoral tissues to the fixation process, leading to more pronounced morphological alterations. For global morphology, a parameter assessing overall tissue morphology, tumoral tissues exhibited a higher incidence of lower scores, indicating compromised tissue morphology under fixation. Normal tissues demonstrated a trend towards higher global morphology scores, implying better preservation and resilience to fixation-induced changes.

According to Bass et al. [14], a systematic review that enhances the evidence on the impact of pre-analytical factors in tissue analysis was also consistent with our findings. Their meticulous investigation of multiple articles shows how cold ischemia and fixation time, among others, can have significant associations with degradation on the outcomes of molecular, proteomic, and morphologic analysis of FFPE tissues. Our study’s results are also aligned with this point, emphasizing the critical importance of cold ischemia time in preserving tumoral tissues’ morphological integrity, especially for digestive cancers. Bass et al.’s review strengthens even further why adherence to optimal pre-analytical conditions is a must for effective research on tissue specimens.

The research conducted by Moatamed et al. [18] illustrates a major cornerstone in the field of pathology, that preanalytic variables play a significant role and can significantly impact the diagnostic test results. Despite using a different set of parameters, they have confirmed that the accuracy of HER2/neu in breast cancer remains beyond recommended preanalytic variables with some exceptions. This result is also consistent with ours, which suggests that cold ischemia time has a more pronounced influence while fixation time also plays a role, though to a lesser extent, on tissue morphology. On the subject of cold ischemia, Thaer Khoury [19] reviewed its impact on breast cancer markers. An interesting fact here is that cold ischemia for long periods can influence the manifestation and valuation of definite biomarkers, a result we further investigated in gastrointestinal malignancies. The study strongly suggests that cold ischemic times should be taken into account when choosing a marker to evaluate it as they can seriously distort a biomarker’s representation, which applies to all cancer types.

The research by Heatley [20] contributes to our investigation, with the conclusion that the temperature and the technique used for storing tissues following excision; to a larger extent, fixation influences the maintenance of tissue morphology and structure. The revelations of Heatley supported by our findings also underscore the imperative nature of pre-analytical factors in histopathological analysis. In agreement with our results, these studies emphasize the need for careful regulation of preanalytic factors, particularly the time of cold ischemia, to guarantee the validity and precision of pathological examinations when applying them in cancer diagnostics [21]. The pool of information confirms not only our observations but also points out the wider relevance of these parameters in oncology research and diagnostics [22].

Clinical recommendations based on study findings: Based on our data, we propose the following evidence-based guidelines:

1. Pancreatic and liver specimens should be processed within 3–6 h to maintain diagnostic quality;
2. Cold ischemia time should be recorded from vascular clamping, not specimen removal;
3. Fixation should not exceed 96 h to preserve immunohistochemical reactivity;
4. Organ-specific handling protocols should be implemented with stricter constraints for enzymatically active tissues.

## 5. Conclusions

The main conclusion that can be drawn from our study is that cold ischemia time (CIT) and fixation time (FT) have significant associations with degradation effects on digestive cancer tissues, but these impacts are lesser on normal tissues. Tumoral tissues were found to be subjected to a high degree of morphological deterioration after prolonged CIT and FT, suggesting the high vulnerability of cancerous tissue to these pre-analytical conditions. Normal tissues also demonstrated a susceptibility towards the fixation processes which mainly affected membrane integrity and nucleic features.

The findings can be a valuable source for further investigations of digestive cancer because they provide a basis for future studies and an opportunity to perform even more extensive research to better understand how these pre-analytical factors affect other aspects of malignant tissues, like the constancy of molecular profiles and the prevalence of pre-analytical factors.

## Figures and Tables

**Table 1:** Study protocol design.

Normal tissues									tumoral Tissues								
↓									↓								
Nine Fragments									Nine Fragments								
N = 5					N = 4				N = 5					N = 4			
↓					↓				↓					↓			
Cold ischemia time (CIT)					Fixation time (FT)				Cold ischemia time (CIT)					Fixation time (FT)			
24 h of fixation					1 h of CIT				24 h of fixation					1 h of CIT			
↓	↓	↓	↓	↓	↓	↓	↓	↓	↓	↓	↓	↓	↓	↓	↓	↓	↓
CIT-1	CIT-2	CIT-3	CIT-4	CIT-5	FT1	FT2	FT3	FT4	CIT-1	CIT-2	CIT-3	CIT-4	CIT-5	FT1	FT2	FT3	FT4

**Table 2:** Scoring system.

Parameter	Good (2)	Fair (1)	Bad (0)
**Morphology**	Clear architectural preservation, distinct tissue boundaries, intact epithelial–stromal interface	Moderate architectural distortion, partially obscured boundaries, recognizable structure	Severe architectural loss, unrecognizable boundaries, complete structural disintegration
**Cytoplasmic details**	Sharp cytoplasmic borders, clear organelle definition, uniform staining	Moderate cytoplasmic blurring, partial organelle visibility, mild vacuolization	Complete cytoplasmic dissolution, no organelle visibility, extensive vacuolization
**Nuclear details**	Crisp nuclear membranes, clear chromatin pattern, distinct nucleoli	Mild nuclear smudging, partially visible chromatin, reduced nucleolar clarity	Complete nuclear degradation, chromatin clumping, nuclear pyknosis
**Membrane**	Intact membrane continuity, sharp delineation, preserved basement membrane	Partial membrane disruption, focal discontinuity, mild retraction	Complete membrane loss, extensive fragmentation, severe retraction artifacts

## Data Availability

The original contributions presented in this study are included in the article. Further inquiries can be directed to the corresponding authors.
